# Analysis of IgE level influencing factors of common allergens for allergic rhinitis in northeastern China

**DOI:** 10.1016/j.clinsp.2024.100495

**Published:** 2024-09-11

**Authors:** Yanling Chu, Ai-Hui Yan

**Affiliations:** a Department of Otolaryngology, The Quzhou Affiliated Hospital of Wenzhou Medical University, Quzhou People's Hospital, Quzhou, China; b Department of Otolaryngology, The First Hospital of China Medical University, Shenyang, China

**Keywords:** IgE level influencing factors, Allergic rhinitis (AR), Allergen, Preventive basis, Therapeutic basis, Allergenic factors

## Abstract

**Objectives:**

This thesis aims to provide patients with a preventive and therapeutic basis by analyzing IgE level influencing factors of common allergens for Allergic Rhinitis (AR).

**Method:**

Multiple linear regression analysis is made upon questionnaires among 749 cases of AR patients that are divided into 5 age-based groups. Perform serum-specific IgE content testing on patients.

**Results:**

Cockroach being an allergen, AR patients’ IgE Level is influenced by allergic history, home-raised plants and animals. For AR patients with mugwort as an allergen, allergy and asthma history could increase IgE level, respectively, β = 4.291 and β = 4.364. If the allergen turns out to be peanut, allergic history would increase the IgE level (β = 0.171), however, the level would be lower in female patients compared with male patients (β = -0.078). For patients with egg as an allergen, allergic history, home-raised plants and animals (pets) would all affect the IgE level, respectively, β = 0.182, β = 0.118 and β = -0.101.

**Conclusions:**

IgE level varies according to allergic history, home-raised plants & animals, gender, furniture renewal, asthma, and ages for patients with different allergens including cockroach, mold, mugwort, peanut, egg and crab. For each kind of allergen, the IgE levels react differently to different influencing factors, thus requiring a thorough analysis of each AR patient's allergen and allergenic factors.

## Highlights

• The IgE level is lower in female patients with AR compared with male patients.
• AR patients’ IgE Level is affected by allergic history, home-raised plants, and pets.
• IgE levels vary with furniture renewal, asthma, and age in allergic rhinitis patients.

## Introduction

Allergic Rhinitis (AR) is a chronic inflammatory disease in the nasal mucosa. It occurs when an allergen invades an individual with a predisposition, triggering the IgE to bind a series of cytokines and immunocompetent cells to produce a large number of histamine.^1^ Among the common diseases in the otolaryngology department, AR sees a relatively high morbidity, which is also a worldwide non-infective but chronic disease, whose annual average of prevalence rate reaches 10 %‒25 %.^2^ AR prevalence witnesses an annual rise in many Chinese medium and large cities, severely affecting patients’ health, both physically and psychologically. In return, this disease has more or less hindered social and economic development. In the USA, UK, Finland and Switzerland, AR morbidity is 10 %‒20 % among the population,^3^ and China also experiences a rising trend with the disease. Change of human hereditary susceptibility alone fails to explain this phenomenon. Environmental factors have a significant impact on allergic rhinitis, including pollen, dust mites, mold, pet dander, air pollutants, etc. These factors can cause allergic reactions, leading to the onset of allergic rhinitis. These environmental factors can stimulate the human immune system, leading to allergic reactions, including tissue inflammation and excessive release of substances such as histamine, which can cause symptoms such as nasal mucosal congestion, increased secretion, and sneezing. Currently, however, the action mechanism of their interrelationship has not been elaborated by researchers in this field.

## Materials and methods

### Research subjects

Subjects of this thesis cover 749 AR patients at China Medical University from Aug 2017 to May 2018, involving 501 males (66.8 %) and 248 females (33.2 %). The patients’ ages vary from 6 to 65 years old and are divided into 5 groups: Group A (≤ 6 years-old), Group B (7‒14 years-old), Group C (15‒35 years-old), Group D (36‒60 years old) and Group E (61‒65 years old). Questionnaires are carried out among these subjects. This study was approved by the Ethics Committee of The First Hospital of China Medical University (n° 2017–014) and followed the STROBE Statement. All patients have signed an informed consent form.

### Exclusion criteria

Excluded subjects cover those who have nasal polyps, nasal tumors and obvious deviation of nasal septum after examination. Patients with flu and infection symptoms are not counted in the subjects and those with incomplete investigation data are also excluded.

### Data collection

Questionnaires mainly survey the patients’ basic information, including their names, gender, age, occupation, allergic history, family genetic disease, asthma, food allergy, smoking, dust inhalation, and exposure to automobile exhaust, home-raised plants, animals, short-term decoration and furniture renewal.

### Test method

#### Blood serum-specific IgE test content

The test strip contains 8 kinds (20 categories) of food allergens and 7 kinds (14 categories) of inhaled allergies.

Food allergens are mutton, beef, milk, scallop/shrimp/crab, weever/cod/salmon, egg yolk/egg white, peanut/cashew/pistachio/hazel, mango/apple/peach/pineapple/strawberry.

Inhaled allergens are dermatophagoides pteronyssinus/dermatophagoides farinae, mugwort, ragweed, cypress/elm/willow/populus, mold/, cladosporium/aspergillus fumigatus/mucor, cat and dog dander.

### Test equipments and method

The test equipment applied in this thesis are as follows: In Vitro Allergen Determination System (Allergy Screen made by Mediwiss, Germany), Immunoblotting Technique, Decolorizing Transfer Table, Rapid Reader, Special Cassette, Allergen Test Strip and supporting reagents.

### Statistical analysis

Statistics work such as setting up database and statistical analysis was done with SPSS 22.0. Multiple comparisons are carried out according to LSD-t-test and multiple linear regression analysis is made to identify how AR patients’ IgE levels are influenced by different factors. The hypothesis test is two-sided, and the inspection level is set as α = 0.05.

## Results

### Influence of inhaled allergen on IgE level

#### IgE level influencing factors for patients with cockroaches as an allergen

Cockroach being an allergen, AR patients’ IgE Level is influenced by allergic history, home raised plants and animals, which, in total, could explain 9.7 % of the IgE level changes (R^2^ = 0.097). Allergy history could increase the IgE level (β = 0.168), and home-raised plants could also increase IgE level (β = 0.148), but animals’ influence shows a decrease in IgE level (β = −0.171). See Table 1.

**Table 1:** IgE level influencing factors for patients with cockroach as an allergen.

Factors	β	*t*	p
Gender	−0.070	−1.111	0.268
Age	0.007	0.138	0.890
Occupation	−0.003	−0.259	0.796
Allergic history	0.168	1.975	0.049
Family genetic disease	0.087	1.460	0.146
Asthma history	0.005	0.072	0.943
History of diet allergy	0.078	0.684	0.494
Smoking	−0.014	−0.326	0.744
Dust exposure	0.014	0.191	0.849
Automobile exhaust	−0.108	−1.485	0.139
Home raised plants	0.148	2.439	0.015
Home raised pets	−0.171	−2.798	0.006
Short-term decoration	0.131	0.846	0.398
Furniture renewal	−0.187	−1.581	0.115

### IgE level influencing factors for patients with mugwort as an allergen

For patients with mugwort as an allergen, allergy and asthma history could increase IgE levels, respectively, β = 4.291 and β = 4.364. These could account for 8.2 % (R^2^ = 0.082) of the IgE level changes. See Table 2.

**Table 2:** IgE level influencing factors for patients with mugwort as an allergen.

Factors	β	*t*	p
Gender	−0.020	−0.019	0.985
Age	0.743	0.852	0.395
Occupation	0.308	1.332	0.184
Allergic history	4.291	2.727	0.007
Family genetic disease	−0.472	−0.446	0.656
Asthma history	4.364	3.675	0.000
History of diet allergy	−1.187	−0.603	0.547
Smoking	0.026	0.035	0.972
Dust exposure	−1.119	−0.819	0.413
Automobile exhaust	−2.396	−1.785	0.075
Home raised plants	0.958	0.933	0.352
Home raised pets	−1.168	−1.073	0.284
Short-term decoration	2.405	0.959	0.338
Furniture renewal	−0.650	−0.330	0.742

### Influencing factors for food allergens

#### IgE level influencing factors for patients with peanut as an allergen

If the allergen turns out to be peanut, gender and allergic history would both affect the IgE level. Allergic history can increase the level (β = 0.171), while it would be lower in female patients compared with male patients (β = −0.078). These two factors could explain 7.4 % of the IgE level changes (R^2^ = 0.074). See Table 3.

**Table 3:** IgE level influencing factors for patients with peanut as an allergen.

Factors	β	*t*	p
Gender	−0.078	−2.088	0.038
Occupation	−0.010	−1.265	0.207
Age	−0.008	−0.263	0.793
Allergic history	0.171	3.042	0.003
Asthma history	−0.003	−0.069	0.945
History of diet allergy	−0.028	−0.398	0.691
Smoking	−0.019	−0.716	0.475
Dust exposure	0.014	0.289	0.773
Automobile exhaust	0.006	0.122	0.903
Home raised plants	0.052	1.418	0.157
Home raised pets	−0.044	−1.144	0.253
Short-term decoration	0.087	0.966	0.335
Furniture renewal	0.013	0.180	0.857

#### IgE level influencing factors for patients with egg as an allergen

For patients with egg as an allergen, allergic history, home-raised plants and animals (pets) would all affect the IgE level, respectively, β = 0.182, β = 0.118 and β = −0.101. These would contribute to 8.3 % of the IgE level changes (R^2^ = 0.083). See Table 4.

**Table 4:** IgE level influencing factors for patients with egg as an allergen.

Factors	β	*t*	p
Gender	0.003	0.090	0.928
Age	0.011	0.342	0.733
Allergic history	0.035	0.602	0.548
Allergic history	0.047	1.212	0.226
Asthma history	0.035	0.801	0.423
History of diet allergy	0.182	2.535	0.012
Smoking	−0.046	−1.707	0.089
Dust exposure	−0.010	−0.209	0.835
Automobile exhaust	−0.061	−1.246	0.214
Home raised plants	0.118	3.139	0.002
Home raised pets	−0.101	−2.544	0.011
Short-term decoration	−0.006	−0.063	0.950
Furniture renewal	0.000	0.054	0.957

#### IgE level influencing factors for patients with crab as an allergen

As an allergen, crab influence on IgE level is affected by allergic history and home-raised animals, the former would increase the level (β = 0.291), while the latter would lower the level (β = −0.137). Allergic history and crab, in total, would bring about 9.2 % of the level changes. See Table 5.

**Table 5:** IgE level influencing factors for patients with crab as an allergen.

Factors	β	*t*	p
Gender	−0.074	−1.149	0.252
Age	0.032	0.586	0.559
Allergic history	0.291	3.310	0.001
Family genetic history	0.047	0.762	0.447
Asthma history	0.089	1.335	0.183
History of diet allergy	−0.101	−0.858	0.392
Smoking	0.044	0.966	0.335
Dust exposure	−0.075	−0.951	0.343
Automobile exhaust	−0.059	−0.776	0.439
Home raised plants	0.085	1.349	0.179
Home raised pets	−0.137	−2.170	0.031
Short-term decoration	0.024	0.147	0.883
Furniture renewal	0.000	0.000	1.000

## Discussion

Regional epidemic disease data of AR is of great importance.^4^, ^5^, ^6^ Early prevention and diagnosis could significantly reduce AR prevalence and improve the patient's life quality.^7^, ^8^, ^9^ Past epidemiological research mainly employed multiple linear regression methods to discuss the possible factors that influence the IgE level,^10^ and then conducted a layering study of different allergens. Listing out some common allergens such as cockroach, mugwort, peanut, egg, and crab, in contrast, this thesis conducts a multiple linear regression analysis of each factor's influence on IgE level. The result reveals that, for different allergens, there exists a remarkable difference in IgE level influencing factors. This also indicates that risk factors do not remain the same for AR that is triggered by different allergens.

### Characteristics of influencing factors of cockroach as an allergen

Cockroach being an allergen, IgE Level is influenced by allergic history, home raised plants and animals, which, in total, could explain 9.7 % of the IgE level changes (R^2^ = 0.097). Allergy history could increase the IgE level (β = 0.168) and home-raised plants could also increase the IgE level (β = 0.148), but animals’ influence shows a decrease in IgE level (β = −0.171). IgE concentration could provide a reference for diagnosis on the one hand, and it could reflect the patients’ allergic severity on the other hand.^11^ The cockroach is one of the factors that causes AR.Lab test shows they carry over 40 kinds of bacteria including salmonella, dysentery bacterium, aspergillus flavus, and 7 kinds of parasitic ovum such as roundworm and hookworm.^12^^,^^13^ Cockroaches are generally omnivorous. They would spit out their stomach secretions first and suck them back together with food. Their special way of feeding can easily contaminate human food and cooking utensils, thus spreading many diseases like dysentery, typhoid, hepatitis and causing allergic symptoms. In general, cockroaches shelter in shade corners, mainly in the kitchen and drainage pipelines. To prevent AR triggered by cockroaches, one should keep a clean living environment and stay away from this allergen.

### Analysis of the influence of allergic history on IgE level for AR patients with cockroaches as an allergen

Allergy history could increase the IgE level (β = 0.168) for patients with cockroaches as the allergen. When an individual with a predisposition gets exposed to an allergen, the IgE will be triggered to bind a series of cytokines and immunocompetent cells to produce a large number of histamines, causing AR, a noninfectious inflammatory disease in the nasal mucosa. The happening of AR has two requirements, one being a specific antigen that leads to immunoreactions, the other being an individual with predisposition, or allergic constitution. Once the two conditions mix, AR symptoms will occur. AR hypersensitivity is essentially an immunologic derangement that is mainly caused by an individual's susceptibility of the immune system to allergens. With the first exposure history to a certain allergen, one's second exposure to the same allergen would trigger a stronger immune response, leading to inflammation and a series of other clinical symptoms. If a cockroach is an AR patient's allergen, his IgE level would increase should he have another allergic history, either drug, food, or contact. As an AR predisposing factor, allergic history has a broader impact on IgE levels for patients with different allergens. This thesis concludes that allergic history influences the patients’ IgE level if the allergens are inhaled like cockroach, mold, and mugwort, and intake ones like peanut and crab.

### Analysis of the influence of home-raised plants on IgE level for AR patients with cockroaches as an allergen

Cockroach being the allergen, home-raised plants would IgE level (β = 0.148). AR that is caused by pollens is nicknamed pollinosis, an allergic disease on the nasal mucosa. If one has a pollens allergic history, his following exposures to pollens would trigger the antigens and antibodies to combine and release histamine and leukotrienes. Pollens are tiny reproductive male cells from flowers, which, after ripening, will drift in the air and spread as far as 400 miles. During a flowering season, pollens, mingled with automobile exhaust and industrial particles, though invisible, would drift with the wind and spread everywhere, exposing AR patients to this harassing allergen. The reason for discomfort caused by pollens lies in that they contain abundant macromolecular vegetable protein, which, once inhaled into the respiratory tract by an individual with a predisposition, will generate a train of antigen-antibody reactions. These reactions are as follows: allergens like pollens and mold enter the nasal cavity and weasand→stimulate the body to produce a special antibody IgE→IgE combines with mast cells→allergens enter human bodies again→mast cells release histamines→outburst of allergic symptoms. For patients with cockroaches as allergen, should they have home-raised plants, capable of blooming in particular, they would have a higher IgE level and suffer from more severe symptoms compared with those who are plant-free at home. However, no research reports, neither from domestic nor overseas, can be found in this field.

### Analysis of the influence of home-raised pets on IgE level for AR patients with cockroaches as an allergen

Home-raised pets could lower the AR patients’ IgE levels with cockroaches as the allergen (β = −0.171). Nowadays, people's life quality is improving rapidly, and people are no longer confined to material needs. More and more pets are raised at home to meet people's spiritual needs. The most common pets are cats and dogs. Research shows, in the general population, if one is allergic to pets, diseases like AR, allergic asthma, allergic conjunctivitis, nettle rash, and specific eczema can occur after he inhales or has skin exposure to pets’ danders. Allergens from cats and dogs, which might drift in the air for a long time, can be easily inhaled to the human's respiratory tract and cause allergic diseases.

Clothing is the main carrier of pet allergens, one's exposure to which, will stain clothing and turn them into allergen carriers. The allergens are brought by these people from homes to public places and office areas, causing allergic symptoms among those people who are vulnerable to pets. This also explains why someone does not raise pets at home, but experiences pets' allergic symptoms. Allergy is becoming a common and frequently occurring disease. Staying away from allergens is the best treatment for allergies.

Home-raised pets could lower AR patients’ IgE levels with cockroaches as the allergen. This is an unexpected result in the research, which might contribute to a cleaner environment in houses with pets. Compared with pets-free houses, areas providing shelter to cockroaches in the house with pets tend to be cleaned more frequently, decreasing the cockroaches’ reproduction ability, thus lowering the IgE level. Patients’ allergic symptoms could be accordingly slighter. But research in this field needs to be further studied.

### Characteristics of influencing factors on IgE level with mugwort as an allergen

As a common allergen in the north of the Yangtze River, mugwort usually enjoys a long pollination period and shows strong reproduction ability. Mugwort is planted in some cities for urban greening, but it is recommended that governments of all levels switch to other alternative greening plants with less strong antigenicity, fewer pollens and lower sensitization rate.^14^ This thesis also finds that ragweed pollen, showing seasonal and regional characteristics, is another threat to AR patients’ health in Shenyang, and its positive rate reaches 13.4 %.

For patients with mugwort as an allergen, allergy and asthma history could increase IgE levels, respectively, β = 4.291 and β = 4.364. These could account for 8.2 % (R^2^ = 0.082) of the IgE level changes.

### Analysis of the influence of allergic history on IgE level for patients with murwort as an allergen

Allergic history could raise the IgE level (β = 4.291). The mechanism and correlation are the same as discussed in previous section 4.1.1.

### Analysis of the influence of asthma on IgE level for patients with murwort as an allergen

This research finds that asthma history could increase IgE level (β = 4.364). On AR and asthma patients’ mucous membrane and air passage, there is a rising number of Th2 cells that produce specific immune factors and trigger allergic reactions by releasing large amounts of tryptase. A rise of the expression level of Intercellular Adhesion Molecule 1 (ICAM-1) and other ICAMs is found at last on the airway epithelium. Through flow cytometry and polymerase chain reaction, Yam amoto^15^ discovered that a certain amount of NK cells could be found on the mucous membrane of asthma patients, but these kinds of cells do not exist on the nasal mucosa of AR patients and healthy people. In addition, researchers find there exist many Th2 cells on the nasal mucosa of asthma patients, while the number of IFN and Th1 cells remains unchanged. This reveals that, for asthma patients, NK cells are capable of producing and infiltrating Th2 cells without relying on the triggering of antigens. When an individual is in healthy condition, the Th1/Th2 cells rest in a steady state, allowing the body to function as effective immune defense. Similarly, when an AR patient with an allergic history receives stimulations of mugwort, his Th1/Th2 cells will lose the balanced state, which would prevent the immune system from functioning effectively, leading to the increase of IgE level at last. An overseas study points out, that, as an inflammatory T-cell, Th17 can mediate the inflammation of T-cells and infiltration of granulocytes during an airway inflammation progress. However, researchers fail to elaborate on whether determining the T-cell is helpful for better clinical treatment of AR.

### Characteristics of influencing factors on IgE level with egg as an allergen

In this study, allergic history, home-raised plants and pets would all affect the IgE level of patients with egg as an allergen. The mechanism of how food allergens cause AR is as follows: food allergens could lead to immediate fatal hypersensitivity by triggering the intestinal mucosa cells to produce high concentration medium. A lab test of the gastric juice, after receiving antigen stimulation, shows that there is a close relationship between the antigenicity of food and its anti-digestibility in the gastrointestinal tract. Some antigens have enzymatic functions. For instance, one sort of antigen, besides its close relationship with pediatric asthma, is able to split up into CD23 and CD25 which exist in B-cells. With a weak ability to integrate with IgE antibody, CD23 can easily generate abundant IgE. Lack of antibodies integrating IgE on B-cells would induce T-cells to overreact their immunologic effect to Th2 hypersensitivity. In this study, patients’ IgE level is affected by allergic history, home-raised plants and pets. These influencing factors could contribute to 8.3 % of the IgE level changes (R^2^ = 0.083). Compared with other common allergic histories, food allergic history can better explain the influence of food allergens on IgE levels. The influencing mechanisms of home-raised plants and pets rest the same as discussed above.

### Characteristics of influencing factors on IgE level with peanut as an allergen

For patients with peanuts as an allergen, the IgE level will be influenced by gender and allergic history. These two factors could explain 7.4 % of the IgE level changes (R^2^ = 0.074). For gender considerations, being female is a protective factor, which might be due to the male pressure and their smoking, diet and alcohol-drinking habits. Allergic history could increase the IgE level (β = 0.171). When an individual with an allergic history has a second exposure to the same allergen, his body will present a stronger immune response, which causes inflammation and other clinical symptoms. The mechanism is the same as discussed in previous section 4.1.1

### Characteristics of influencing factors on IgE level with crab as an allergen

As an allergen, crab influence on IgE level is affected by allergic history and home-raised pets, which bring about 9.2 % of the level changes. According to research reports, there are rich seafood resources in coastal cities like ShenZhen and Qingdao, where more AR patients are seen with seafood as their allergen. This is solid proof of the close relationship between food allergens and dietary structure. To avoid AR, one should take necessary protective measures to stay away from the inhaled allergens and refrain from eating or even having skin contact with those reported food allergens. In addition, determining food allergens, on the one hand, is helpful for the clinical diagnosis of allergies from an etiologic perspective, and on the other hand, it would benefit relevant research on the development mechanism of AR. As a popular food on the dining table in north China, crabs have caused an increasing number of people's allergies. Allergic history can increase the IgE level and home-raised pets can decrease its level. This conclusion is of great significance for patients to avoid allergens and exclude the risk factors.

Through non-conditional logistic regression analysis, Pang Yufeng^16^ carried out research among 150 cases of AR and 150 cases of controls. He concludes that asthma history, AR family history, <7 h of daily sleeping time, keeping pets, and working in formidable conditions with much dust are the risk factors that are related to the occurrence of AR. Another research adopts a stratified surveying method to analyze the epidemiological data of AR and finds that its prevalence rate is higher among youth groups, but the rate will drop with age.^17^

## Conclusion

In summary, due to the influence of various risk factors, the research conclusions of AR in different regions are not entirely the same. This article found that the IgE levels of AR patients in the Northeast region vary depending on their allergy history, domesticated flora and fauna, gender, furniture updates, asthma, and age. Clinical needs to thoroughly analyze the allergens and allergenic factors of each AR patient, and then provide them with tailored prevention and diagnostic treatment measures.

## Authors’ contributions

Yanling Chu: Conceptualization; investigation; data curation; formal analysis, validation; writing-original draft; writing-review & editing.

Ai-Hui Yan: Conceptualization; investigation; data curation; formal analysis; validation; writing-review & editing.

## Funding

This research did not receive any specific grant from funding agencies in the public, commercial, or not-for-profit sectors.

## Conflicts of interest

The authors declare no conflicts of interest.
